# Association between loneliness and psychological distress: A cross-sectional study among Japanese workers during the COVID-19 pandemic

**DOI:** 10.1016/j.pmedr.2021.101621

**Published:** 2021-10-25

**Authors:** Yusuke Konno, Masako Nagata, Ayako Hino, Seiichiro Tateishi, Mayumi Tsuji, Akira Ogami, Reiji Yoshimura, Shinya Matsuda, Yoshihisa Fujino

**Affiliations:** a Department of Environmental Epidemiology, Institute of Industrial Ecological Sciences, University of Occupational and Environmental Health, Japan, Kitakyushu, Japan; b Department of Psychiatry, University of Occupational and Environmental Health, Japan, Kitakyushu, Japan; c Department of Occupational Health Practice and Management, Institute of Industrial Ecological Sciences, University of Occupational and Environmental Health, Japan, Kitakyushu, Japan; d Department of Mental Health, Institute of Industrial Ecological Sciences, University of Occupational and Environmental Health, Japan, Kitakyushu, Japan; e Department of Occupational Medicine, School of Medicine, University of Occupational and Environmental Health, Japan, Kitakyushu, Japan; f Department of Environmental Health, School of Medicine, University of Occupational and Environmental Health, Japan, Kitakyushu, Japan; g Department of Work Systems and Health, Institute of Industrial Ecological Sciences, University of Occupational and Environmental Health, Japan, Kitakyushu, Japan; h Department of Preventive Medicine and Community Health, School of Medicine, University of Occupational and Environmental Health, Japan, Kitakyushu, Japan

**Keywords:** COVID-19, Loneliness, Psychological distress, Japan, Worker

## Abstract

The purpose of this study was to examine the association between loneliness and psychological distress during the COVID-19 pandemic in Japan. We conducted a cross-sectional online study from 22 to 26 December 2020. A total of 27,036 participants, all employed at the time, were included in the analysis. Participants were asked if they felt loneliness in a single-item question. The Kessler 6 (K6) was used to assess psychological distress, defined as mild for K6 scores of 5 to 12 and severe for 13 or higher. The odds ratios (ORs) of psychological distress associated with loneliness were estimated using a multilevel logistic model nested in the prefecture of residence, with adjustment for age, sex, marital status, equivalent income, educational level, smoking, alcohol consumption, job type, number of workplace employees, and cumulative incidence rate of COVID-19 in the prefecture. Communication with friends, acquaintances, and family was strongly associated with psychological distress, so we adjusted for these factors and eating meals alone. Results showed a significant association between loneliness and psychological distress (OR = 36.62, 95% CI = 32.95–40.69). Lack of friends to talk to, lack of acquaintances to ask for help, and lack of people to communicate with through social networking sites were all strongly associated with psychological distress, as were family time and solitary eating. Even after adjusting for these factors, loneliness remained strongly associated with psychological distress (OR = 29.36, 95% CI = 26.44–32.98). The association between loneliness during the COVID-19 pandemic and psychological distress indicates the need for intervention.

## 1 Introduction

The outbreak of coronavirus disease 2019 (COVID-19) caused by SARS-CoV-2 was first reported in Wuhan, China, in December 2019, and it has since spread around the world. The World Health Organization (WHO) declared a public health emergency on 30 January 2020, and on 11 March 2020, it declared COVID-19 a pandemic. In Japan, the first case was reported on 16 January 2020, and the disease was designated as a designated infectious disease on 1 February 2020. As of March 2021, the disease continues to spread around the world. Japan is no exception. Since the first case was reported, the outbreak has spread, with a cumulative total of 324,846 confirmed cases in the year from 16 January 2020 to 16 January 2021 (Outbreaks in Japan). In addition to its infectiousness, COVID-19 was found to be highly contagious, with a high severity of illness and a high mortality rate in severely ill patients, leading to measures aimed at preventing COVID-19 in many countries around the world (Weiss and Murdoch, 2020).

In its “Coronavirus disease (COVID-19) advice for the public” to combat COVID-19, WHO advised to: “Maintain at least a 1-meter distance between yourself and others to reduce your risk of infection when they cough, sneeze or speak. Maintain an even greater distance between yourself and others when indoors.” It also recommends avoiding the 3Cs (i.e., spaces that are closed, crowded, or involve close contact), using the phrase “Avoid the 3Cs” (World Health Organization, 2021).

These COVID-19 measures were also applied in Japan, resulting in a transformation of lifestyles and making people more isolated. The Ministry of Health, Labour and Welfare (MHLW) has announced a “new lifestyle” for the new coronavirus, recommending that people avoid contact with other people as much as possible, and if they do make contact, it should be for short periods of time; they should avoid talking and maintain as much distance from others as possible. People are advised to refrain from going out unnecessarily and to shop online as much as possible (Ministry of Health, Labour and Welfare). Living the kind of life recommended for COVID-19 prevention limits communication and makes it easier to socially withdraw.

The work environment is no exception with respect to COVID-19 measures, resulting in greater isolation. “Examples of ‘new lifestyle’ practices in anticipation of the new coronavirus” recommend telework and online meetings, rota working, and staggered commuting (Ministry of Health, Labour and Welfare). As a result, employees work alone at home and meetings are held on the web. They no longer have dinners with their workmates but rather eat and drink alone. No more crowded commuting; no more being with others in enclosed spaces, such as offices or conference rooms; and no more conversations without wearing a mask when eating or drinking. In parallel, opportunities for both public and private communication have decreased, and people’s lifestyles have become more solitary than before the COVID-19 pandemic. People became socially isolated and felt lonely more easily. Loneliness and social isolation are similar, but loneliness is subjective and social isolation is objective (Wheeler et al., 1983). Thus, loneliness is associated with psychological problems, such as depression and anxiety, and social isolation is considered to be a predictor of loneliness (Sanders, 2020). Loneliness is considered to have a negative impact on physical and mental health. A survey conducted among US adults in the early stages of the COVID-19 pandemic reported that loneliness was higher than before the pandemic (Rosenberg et al., 2021). A survey conducted in Wuhan also showed that loneliness among people was increasing in prevalence before the COVID-19 pandemic (Torales et al., 2020). In sum, during the current pandemic, loneliness among people has been increasing.

It has been reported that, during the COVID-19 pandemic, people have more isolated lives and that feelings of psychological distress have increased due to COVID-19 prevention measures (Qiu et al., 2020, Wang et al., 2020). In France, for example, mental health during lockdown was worse than that before lockdown (Ramiz et al., 2021). In a 2-month follow-up study conducted in Italy during lockdown, stress levels in the general population increased (Roma et al., 2020). Psychological distress causes physical and mental health problems. Physically, psychological distress can trigger the onset and exacerbation of cardiovascular diseases, such as hypertension, arrhythmia, ischemic heart disease and heart failure, and chronic stress can lead to overeating and lack of exercise, which in turn leads to obesity, placing an additional burden on the cardiovascular system (Rosengren et al., 2004, Iso et al., 2002). Furthermore, excessive psychological distress is a risk factor for mental disorders, including depression (Slavich and Irwin, 2014).

The longer the COVID-19 pandemic continues, the more psychological distress is expected to increase, and the more physical and mental disorders caused by psychological distress are expected to increase; therefore, we believe that measures to alleviate psychological distress are necessary. The causes of psychological distress resulting from the COVID-19 pandemic are thought to be diverse, but if one of them is isolated living and the sense of loneliness arises from it, then countermeasures are necessary. The purpose of this study was to examine the association between loneliness and psychological distress in Japanese workers under COVID-19 conditions.

## 2 Methods

### 2.1 Study design and participants

This cross-sectional study was conducted on the Internet from 22 to 26 December 2020. This period covers weekdays of the week before the year-end holidays in Japan. The details of the protocol have already been reported (Fujino et al., 2021). In brief, data were collected from people who were employed at the time of the survey, selected by prefecture, occupation, and gender. Responses received extremely quickly after the survey were opened. Those responses reporting a height below 140 cm or weight below 30 kg and those that were inconsistent across multiple identical questions were excluded as likely to be fraudulent. Invitations to participate were sent via email to monitors registered with Cross Marketing Inc. (Tokyo, Japan). A total of 55,045 registered monitors participated by answering the initial screening questions, and 33,302 monitors responded by matching the survey criteria (worker status, region, gender, and age). Thus, a total of 33,302 people participated. After excluding clearly suspected responses, data from 27,036 participants were included in the analysis. This study was approved by the Ethics Committee of the University of Occupational and Environmental Health, Japan (R2-079).

### 2.2 Assessment of loneliness

In this survey, one question was used to determine whether the participants felt loneliness or not. To the question, “During the last 30 days, how frequently have you felt loneliness?”, the subjects answered by selecting one option from never, a little, sometimes, usually, or always. If the subject answered always, usually, or sometimes, loneliness was considered to be present.

### 2.3 Assessment of psychological distress

The Kessler 6 (K6) was used to assess psychological distress (Kessler et al., 2002). The following six questions were presented to the participants in Japanese: “During the last 30 days, about how often did you feel [nervous/hopeless/restless or fidgety], and [so depressed that nothing could cheer you up/that everything was an effort/worthless]?” The area under the curve (AUC) for the Japanese version of the K6 for the Diagnostic and Statistical Manual of Mental Disorders-IV (DSM-IV) diagnosis of a mood disorder (depression or dysthymia) or anxiety disorder (panic disorder, agoraphobia, social phobia, generalized anxiety disorder, or posttraumatic stress disorder) was 0.94 (95% CI = 0.88–0.99), confirming the validity of the Japanese version of the K6 (Furukawa et al., 2008). In this study, the cutoff for mild psychological distress was a K6 score of 5 to 12, and for severe psychological distress, a score of 13 or higher was used.

### 2.4 Other covariates

The following survey items were considered confounding factors: age, sex, marital status, equivalent income, educational level, smoking, alcohol consumption, job type, number of employees at the workplace and cumulative incidence rate of COVID-19 in the prefecture. The survey also asked questions, such as “Do you have friends or neighbors with whom you can easily engage in small talk or daily conversation?”; “Do you have someone you can ask for help?”; and “Do you have a partner with whom you can communicate closely using SNSs?” The participants responded “Yes” or “No” to these questions. For the question, “Time spent with family having a meal or at home”, participants answered more than 2 h, more than 1 h, more than 30 min, less than 30 min, or almost never. For the question, “How often do you eat all meals of the day alone?”, they answered 6–7 days a week, 4–5 days a week, 2–3 days a week, less than 1 day a week, or hardly ever.

In addition, the cumulative incidence of COVID-19 from the time of the survey to 1 month before in the prefecture of residence was used as a community-level variable. Information was collected from the websites of public institutions.

The confounding factors that we selected for inclusion in the analyses were based on those suggested in previous studies as being related to loneliness and mental health.

### 2.5 Statistical analysis

Odds ratios (ORs) for loneliness and psychological distress were estimated with a logistic model. Psychological distress was defined as mild psychological distress with a K6 score of 5 or higher and severe psychological distress with a K6 score of 13 or higher. In the multivariate model, we adjusted for age, sex, marital status, equivalent income, educational level, smoking, alcohol consumption, job type, number of employees in the workplace, and cumulative incidence rate of COVID-19 in the prefecture. In another model, we added having friends or neighbors with whom to easily make small talk or have daily conversations, having someone who can be asked for a little help, and having a close friend to communicate with on social networking sites. The rate of COVID-19 incidence by prefecture was used as a prefecture-level variable. We further examined the interaction between loneliness and living alone on psychological distress.

A p value of less than 0.05 was considered statistically significant. Stata (Stata Statistical Software: Release 16. College Station, TX: StataCorp LLC.) was used to run the analyses.

## 3 Results

The characteristics of the database are shown in Table 1. Of the 27,036 participants, 2,750 (10%) of them felt loneliness. Of those who reported feeling loneliness, 93.6% had a K6 ≥ 5, and 58.5% had a K6 ≥ 13. In contrast, only 33.9% of the group who did not feel loneliness had a K6 ≥ 5, and 3.5% had a K6 ≥ 13. The percentage of participants who answered “Yes” to the following questions: “Do you have friends or neighbors with whom you can easily engage in small talk or daily conversation?”; “Do you have someone you can ask for help?”; and “Do you have a partner with whom you can communicate closely using SNSs?” was lower in the loneliness group in all cases. Those who spent less time with their family during meals and gatherings were more likely to feel loneliness, while those who ate all their meals alone were more likely to feel loneliness. The percentage of participants who felt loneliness was higher among women, unmarried individuals, and those with low incomes. No differences emerged in relation to job type or number of employees in the workplace.

**Table 1:** Characteristics of participants who have experienced loneliness.

	Non-loneliness	loneliness
	N = 24,286	n = 2,750
Age, mean (SD)	47.3 (10.5)	44.5 (10.1)
Sex, male (%)	12,601 (51.9%)	1,213 (44.1%)

Area		
97–356	4,767 (19.6%)	575 (20.9%)
438–490	4,903 (20.2%)	547 (19.9%)
535–911	4,765 (19.6%)	569 (20.7%)
1168–3496(Non-Kanto)	4,929 (20.3%)	521 (18.9%)
1168–3496(Kanto)	4,922 (20.3%)	538 (19.6%)
Marriage status, married	14,077 (58.0%)	952 (34.6%)

Jobtype		
Mainly desk work	12,132 (50.0%)	1,336 (48.6%)
Mainly work involving communicating with people	6,243 (25.7%)	684 (24.9%)
Mainly labor	5,911 (24.3%)	730 (26.5%)

Equivalent income (million JPY)		
40–249	4,910 (20.2%)	800 (29.1%)
250–375	6,714 (27.6%)	836 (30.4%)
376–499	6,046 (24.9%)	579 (21.1%)
≥500	6,616 (27.2%)	535 (19.5%)

Educational background		
Junior high school	306 (1.3%)	62 (2.3%)
High school	6,190 (25.5%)	763 (27.7%)
University, graduate school, vocational school, junior college	17,790 (73.3%)	1,925 (70.0%)
Current smoke	6,274 (25.8%)	730 (26.5%)

Alcohol consumption		
6–7 days a week	5,179 (21.3%)	495 (18.0%)
4–5 days a week	1,910 (7.9%)	167 (6.1%)
2–3 days a week	2,935 (12.1%)	331 (12.0%)
less than 1 day a week	4,071 (16.8%)	476 (17.3%)
hardly ever	10,191 (42.0%)	1,281 (46.6%)

Number of employees in the workplace		
<10	5,619 (23.1%)	546 (19.9%)
<100	6,183 (25.5%)	757 (27.5%)
<1000	6,379 (26.3%)	774 (28.1%)
>1000	6,105 (25.1%)	673 (24.5%)
Do you have friends or neighbors with whom you can easily engage in small talk or daily conversation?	17,029 (70.1%)	1,057 (38.4%)
Do you have someone you can ask for help?	16,901 (69.6%)	932 (33.9%)
Do you have a partner with whom you can communicate closely using SNSs?	15,032 (61.9%)	1,136 (41.3%)

Time spent with family having a meal or at home		
more than 2 hours	4,103 (16.9%)	272 (9.9%)
more than 1 hour	5,922 (24.4%)	390 (14.2%)
more than 30 minutes	5,160 (21.2%)	451 (16.4%)
less than 30 minutes	3,185 (13.1%)	368 (13.4%)
almost never	5,916 (24.4%)	1,269 (46.1%)

How often do you eat all meals of the day alone?		
6–7 days a week	4,276 (17.6%)	1,026 (37.3%)
4–5 days a week	2,064 (8.5%)	270 (9.8%)
2–3 days a week	2,501 (10.3%)	327 (11.9%)
less than 1 day a week	2,496 (10.3%)	234 (8.5%)
hardly ever	12,949 (53.3%)	893 (32.5%)
k6≥5	8,244 (33.9%)	2573 (93.6%)
k6≥13	852 (3.5%)	16,08 (58.5%)

Table 2 shows the odds ratios (ORs) of loneliness and severe psychological distress estimated by the logistic model. In the age-adjusted model, there was a significant association between loneliness and psychological distress (OR = 37.74, 95% CI = 34.04–41.85). This result was also found in the multivariate analysis (OR = 36.62, 95% CI = 32.95–40.69). Lack of friends to talk to, lack of acquaintances to ask for favors, and lack of people to communicate with through social networking sites were all strongly associated with psychological distress. Family time and solitary eating were both associated with psychological distress. Even after adjusting for these factors, loneliness was still strongly associated with psychological distress (OR = 29.36, 95% CI = 26.44–32.98).

**Table 2:** The association between loneliness and psychological distress.

	Age-sex adjusted				Multivariate*				Multivariate**			
Severe psychological distress (K6≥13)	OR	95% CI		p	OR	95% CI		p	OR	95% CI		p
loneliness	37.74	34.04	41.85	<0.001	36.62	32.95	40.69	<0.001	29.53	26.44	32.98	<0.001
Do you have friends or neighbors with whom you can casually make small talk or have daily conversations?	3.63	3.33	3.96	<0.001	3.40	3.11	3.72	<0.001	1.39	1.21	1.60	<0.001
Do you have someone you can ask for a little help?	4.04	3.70	4.41	<0.001	3.79	3.47	4.15	<0.001	1.59	1.39	1.83	<0.001
I have a close friend who I communicate with on social networking sites.	2.56	2.34	2.79	<0.001	2.40	2.19	2.62	<0.001	1.17	1.03	1.33	0.013

Time spent with family having a meal or at home												
more than 2 hours	reference				reference				reference			
more than 1 hour	1.00	0.86	1.17	0.972	1.01	0.87	1.18	0.866	0.96	0.80	1.15	0.654
more than 30 minutes	1.24	1.07	1.45	0.005	1.20	1.03	1.40	0.021	0.93	0.77	1.13	0.466
less than 30 minutes	1.60	1.35	1.88	0.000	1.47	1.24	1.74	0.000	0.97	0.79	1.19	0.747
almost never	2.19	1.91	2.51	0.000	1.92	1.66	2.23	0.000	0.87	0.71	1.06	0.169

How often do you eat all meals of the day alone?												
6–7 days a week	2.30	2.08	2.55	0.000	1.83	1.68	2.00	0.000	1.10	0.94	1.29	0.217
4–5 days a week	1.76	1.53	2.04	0.000	1.61	1.44	1.80	0.000	1.50	1.24	1.81	0.000
2–3 days a week	1.44	1.25	1.66	0.000	1.48	1.33	1.64	0.000	1.12	0.93	1.34	0.232
less than 1 day a week	1.30	1.12	1.51	0.001	1.35	1.22	1.51	0.000	1.24	1.03	1.49	0.021
hardly ever	reference				reference				reference			

The interaction between living alone and loneliness showed a significant effect (p < 0.001) on psychological distress. Table 3 shows the ORs of loneliness and severe psychological distress according to whether the participant lived alone or not. For those who lived alone and felt loneliness, there was a significant association with the age- and sex-adjusted OR = 43.81 (95% CI = 38.60–49.72) and multivariate analysis OR = 32.47 (95% CI = 28.48–37.02). For those with family or roommates and who felt loneliness, there was a significant association with the age- and sex-adjusted OR = 28.55 (95% CI = 23.26–35.03) and multivariate analysis OR = 23.79 (95% CI = 19.27–29.38).

**Table 3:** Odds ratios for psychological distress associated with loneliness by living arrangement.

	Age-sex adjusted				Multivariate*			
	OR	95%CI		p	OR	95%CI		p
In participants living alone								
loneliness (vs non-loneliness)	43.81	38.60	49.72	<0.001	32.47	28.48	37.02	<0.001
In participants living with family								
loneliness (vs non-loneliness)	28.55	23.26	35.03	<0.001	23.79	19.27	29.38	<0.001

*Multivariate model adjusted for age, sex, marital status, equivalent income, educational level, smoking, alcohol consumption, job type, number of employees at the workplace and cumulative incidence rate of COVID-19 at prefecture, lack of friends to talk to, lack of acquaintances to ask for favors, lack of people to communicate with through social networking sites, family time and solitary eating.

## 4 Discussion

In this study, 10% of all participants felt loneliness, and this loneliness was strongly associated with psychological distress. In a study conducted among frail older adults in the Netherlands during the COVID-19 pandemic, 20% felt loneliness (Kremers et al., 2021). Among older adults in Hong Kong, 8.8% felt loneliness before the COVID-19 pandemic, whereas 27.7% of them felt loneliness during the pandemic (Wong et al., 2020). As the subjects of this study were workers, they necessarily participated in social activities more than the elderly or the general population, and as a result, the percentage of people who felt lonely was lower than in previous studies. When there was social isolation as living alone in addition to loneliness, the OR was higher than for loneliness without social isolation. This suggests that more attention is needed when social isolation is added to loneliness. This means that those living alone were considered to need more attention than those with family or roommates.

Communication with friends, acquaintances, and family was also strongly associated with psychological distress; therefore, we adjusted for spending time with friends, acquaintances, and family, but loneliness was still strongly associated with psychological distress. Loneliness is generally defined as the discrepancy between a person's desired social relationships and their actual social relationships (Russell et al., 1980). Loneliness is considered to be different from social isolation, although there is overlap between the two (Wheeler et al., 1983). In this study, loneliness was assessed by the subjective question, “Have you ever felt loneliness?” In contrast, communication with friends, acquaintances, and family, which was used as an adjustment factor, was assessed using objective questions, as was social isolation. The results of this study showed that loneliness and social isolation overlap, but only partially, and the nonoverlapping parts are considered to be subjective experiences. We believe that this subjective experience is associated with psychological distress and is a factor that should be addressed.

One of the reasons for the loneliness highlighted here is considered to be the particular lifestyle that has emerged due to the COVID-19 pandemic. This lifestyle requires a different way of living, complying with “avoiding the 3Cs” (World Health Organization, 2021) to prevent infection, but as a result, communication has decreased. This has led to a sense of loneliness, which in turn is thought to be linked to psychological distress. As of February 2021, there is no prospect of COVID-19 eradication, and people will need to continue their new lifestyles to prevent infection. Therefore, the incidence of psychological distress is expected to remain high for some time. As severe psychological distress conditions can be physically and mentally disabling, interventions for loneliness are needed if psychological distress under COVID-19 pandemic conditions persists.

As an intervention for loneliness, the usefulness of online communication, such as social media, has been advocated (Seepersad, 2004, Creating, 2015). Online communication tools, such as social networking sites, can be a valuable countermeasure against loneliness during the pandemic and are also useful in terms of infection prevention (Hajek and König, 2021). In a randomized controlled trial conducted during the COVID-19 pandemic, an empathy-focused program of telephone calls significantly improved loneliness (Kahlon et al., 2021). Thus, various types of online communication, such as communication via the web and telephone calls and using social media, may be useful as interventions for loneliness during the COVID-19 pandemic.

However, it has also been pointed out that continual use of social media and other forms of online communication in situations of loneliness may be harmful. One risk is increased psychological distress caused by excessive media consumption. Being inundated with inaccurate information and excessive media consumption during the COVID-19 pandemic may lead to deteriorating mental health (Holmes et al., 2020). The WHO has cautioned that, with the current growing use of social media and the Internet, not only useful but also inaccurate or harmful information about COVID-19 can spread quickly, leading to confusion, health problems and mistrust of health institutions (World Health Organization, 2021).

Another risk is the issue of addiction. Loneliness is a risk factor for substance or behavioral addictions, including the Internet (Hunt et al., 2018, Moody, 2001, Savci, 2016). In addition, addiction to the Internet increases loneliness, and increased loneliness worsens addiction, thus creating a vicious cycle (Bozoglan et al., 2013, Kim et al., 2009).

Thus, there is a risk that online communication, even when used with the intention to reduce psychological distress, may inadvertently exacerbate or cause such distress or lead to addictive behaviors. Online communication is useful as a countermeasure to loneliness, but in some cases, it may have harmful consequences; therefore, it should be used with caution. Infection prevention measures, such as adequate ventilation, keeping a safe physical distance, and using appropriate protective equipment, along with exercise programs, mindfulness, and cognitive-behavioral therapy, all of which are known to be effective in reducing loneliness, may be valuable when time constraints are a consideration (Hwang et al., 2020, Masi et al., 2011).

### 4.1 Limitations of the study

There are several limitations to this study. First, because it was conducted online using the Internet and was targeted at workers, the generalizability of the results is uncertain. Nevertheless, we attempted to minimize participant bias by sampling by occupation, region, and prefecture based on the incidence of infection. Second, there are various ways to assess loneliness (Holt-Lunstad et al., 2010, Holt-Lunstad et al., 2015), but in this study, loneliness was assessed by only asking the question, “Have you ever felt alone?” This method was adapted from previous studies that assessed loneliness with a single question (Courtin and Knapp, 2017). Third, because this is a cross-sectional study, the temporal relationship between loneliness and psychological distress is unknown. Fourth, the effects of loneliness vary by culture and personal factors. For example, there is a culture called “super solo” in Japan, which is characterized by enjoyment of loneliness. In this way, different cultures have their own perceptions of loneliness, which may undermine the external validity of the results of this and indeed some other studies on this topic.

## 5 Conclusion

We found that 10% of the participants felt loneliness living under conditions of the COVID-19 pandemic. Loneliness was associated with psychological distress, which we believe requires intervention. Online communication is considered to be an effective intervention for loneliness, but at the same time, it is important to take into account risks, such as addiction.

## CRediT authorship contribution statement

**Yusuke Konno:** Conceptualization, Formal analysis, Writing – original draft. **Masako Nagata:** Writing – review & editing. **Ayako Hino:** Writing – review & editing. **Seiichiro Tateishi:** Project administration, Funding acquisition, Supervision, Writing – review & editing. **Mayumi Tsuji:** Project administration, Funding acquisition, Supervision, Writing – review & editing. **Akria Ogami:** Project administration, Funding acquisition, Supervision, Writing – review & editing. **Reiji Yoshimura:** Project administration, Funding acquisition, Supervision, Writing – review & editing. **Shinya Matsuda:**: Project administration, Funding acquisition, Supervision, Writing – review & editing. **Yoshihisa Fujino:** Conceptualization, Data curation, Funding acquisition, Investigation, Methodology, Project administration, Resources, Supervision, Writing – original draft, Writing – review & editing.

## Declaration of Competing Interest

The authors declare the following financial interests/personal relationships which may be considered as potential competing interests:

YK, MN, AH, ST, MT, AO, SM, YF report no conflict of interest related to our manuscript. RY has received speaker’s honoraria from Eli Lilly, Janssen, Dainippon Sumitomo, Otsuka, Meiji, Pfizer and Shionogi.
